# The construction of a two-dimensional organic–inorganic hybrid double perovskite ferroelastic with a high *T*_c_ and narrow band gap†

**DOI:** 10.1039/d1sc07045b

**Published:** 2022-03-30

**Authors:** Chang-Yuan Su, Ye-Feng Yao, Zhi-Xu Zhang, Ying Wang, Ming Chen, Pei-Zhi Huang, Yi Zhang, Wen-Cheng Qiao, Da-Wei Fu

**Affiliations:** Ordered Matter Science Research Center, Jiangsu Key Laboratory for Science and Applications of Molecular Ferroelectrics, Southeast University Nanjing 211189 China yizhang1980@seu.edu.cn; Department of Physics, Shanghai Key Laboratory of Magnetic Resonance, School of Physics and Materials Science, East China Normal University Shanghai 200062 China 2023948184@qq.com; Institute for Science and Applications of Molecular Ferroelectrics, Key Laboratory of the Ministry of Education for Advanced Catalysis Materials, Zhejiang Normal University Jinhua 321004 China dawei@seu.edu.cn

## Abstract

Two-dimensional (2D) hybrid double perovskites have attracted extensive research interest for their fascinating physical properties, such as ferroelectricity, X-ray detection, light response and so on. In addition, ferroelastics, as an important branch of ferroic materials, exhibits wide prospects in mechanical switches, shape memory and templating electronic nanostructures. Here, we designed a 2D phase-transition double perovskite ferroelastic through a structurally progressive strategy. This evolution is core to our construction process from 0D to 1D and AgBi-based 2D. In this way, we successfully synthesized 2D lead-free ferroelastic (DPA)_4_AgBiBr_8_ (DPA = 2,2-dimethylpropan-1-aminium) with a high Curie temperature (*T*_c_), which shows a narrower band gap than 0D (DPA)_4_Bi_2_Br_10_ and 1D (DPA)_5_Pb_2_Br_9_. Moreover, the mechanism of structural phase transition and molecular motion are fully characterized by temperature dependent solid-state NMR and single crystal XRD. (DPA)_4_AgBiBr_8_ injects power into the discovery of new ferroelastics or the construction and dimensional adjustment in new hybrid double perovskites.

## Abstract 2

By using a lead-free AgBi-based scheme, we successfully synthesized a two-dimensional double perovskite ferroelastic (DPA)_4_AgBiBr_8_ with high *T*_c_ of 375 K and a narrow band gap of 2.44 eV, where DPA is 2,2-dimethylpropan-1-aminium.

## Graphical abstract

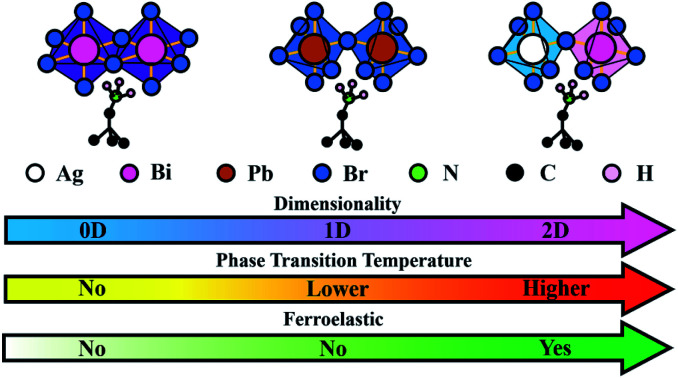

## Introduction

In recent years, lead-based perovskites represented by MAPbI_3_ have swept across numerous scientific research areas including light emitting diodes,^1–5^ ferroelectrics,^6–11^ solar cells,^12–15^ gas sensors,^16^ lasers,^17^ catalyst, photodetectors,^18^ and so on.^19–23^ The excellent features stem from their comparative advantages including high absorption coefficient, high charge-carrier mobility, narrow and tunable bandgaps, and other physical and chemical characteristics in perovskites. However, toxicity and long-term instability of lead-based materials restrict their further development.^24,25^ Its potential negative impacts on animals, plants and the environment are our concerns and need to be overcome. In this research context, lead-free and lead-replacement ones have naturally become new explorations. This is the most direct method for the research and development of lead-free materials. Therefore, a lot of attention is focused here in order to make new scientific breakthroughs.

As a feasible method, homo-valent replacement using Ge and Sn was proposed to construct lead-free perovskite with superior optical and electronic properties.^26–28^ Nevertheless, Ge/Sn-based perovskites have been criticized for their instability, motivating us to try hetero-valent replacement.^29–31^ To maintain charge neutrality, hetero-valent replacement can be divided into two subcategories, namely ion-splitting and ordered vacancies. In the ion-splitting subcategory, mixed cation materials at the B site, with a chemical formula of A_2_B^I^B^III^X_6_, are featured with appropriate electronic dimensionality and rich chemistry, besides their stability, compared to ordered vacancy (A_3_□B^III^X_9_ and A_2_□B^IV^X_6_, □ is vacancy). Here, the B^I^-site cation mainly includes alkali metal and group IB elements, and the B^III^-site cation are abundant elements that can locate at group B and group A, and the X at corner can contain halogen, CN^−^ and NO^3−^.^28–36^ As a member of double perovskites, two-dimensional double perovskites with a formula of A_4_B^I^B^III^X_8_ show multiple fascinating properties, such as ferroelectricity,^37–39^ piezoelectricity,^39^ X-ray detection,^40–42^ light response,^35,36,43^ broad photoluminescence,^44^ phase transition^38^ and so on,^45^ which inspire us to grope new 2D double perovskites with desired properties in lead-free exploration.

**Scheme 1:**
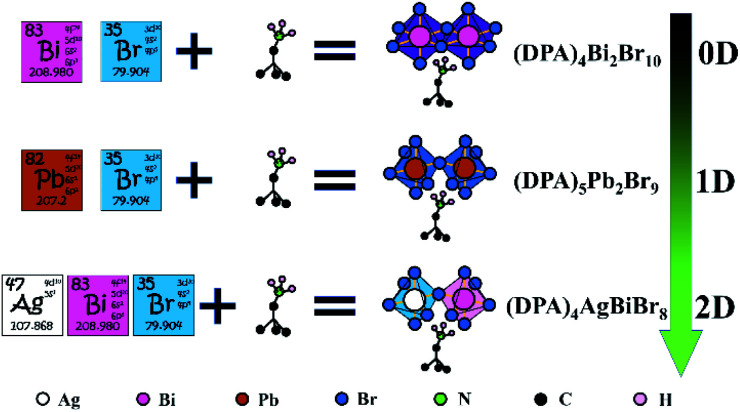
Train of thought from a Bi–Br to Pb–Br to Ag–Bi–Br system based on DPA to realize lead-free hybrid double perovskite.

According to the strategy in Scheme 1: First, we synthesized a zero-dimensional organic–inorganic hybrid compound (DPA)_4_Bi_2_Br_10_, which disappointingly does not show the phase transition or other properties we expected. Then a double-row 1D compound (DPA)_5_Pb_2_Br_9_ with room-temperature phase transition was synthesized by the introduction of Pb^2+^. Based on this construction strategy, finally, the lead-free double perovskite ferroelastic (DPA)_4_AgBiBr_8_ with a high *T*_c_ and narrower band gap was successfully constructed. This is exactly what we expected.

In this work, we deeply explored the structure–activity relationship between structural phase transition and metal substitution. In addition, the single crystal XRD and solid-state NMR were employed to fully characterize the order–disorder characteristics of structural phase transition. As a ferroelastic phase transition material (DPA)_4_AgBiBr_8_ with an Aizu notation of *mmmF*1̄, the transformation of ferroelastic domain structures was clearly observed by using a variable-temperature polarizing microscope. And it is confirmed by UV-vis absorption measurements and density functional theory (DFT) that the band gap of (DPA)_4_AgBiBr_8_ (2.44 eV) is lower than that of (DPA)_4_Bi_2_Br_10_ (2.80 eV) and (DPA)_5_Pb_2_Br_9_ (2.96 eV). In a word, the current report is helpful to the exploration and excavation of more similar lead-free ferroelastics with high temperature phase transformation and hybrid double perovskites.

## Results and discussion

### Basic crystal structure analysis

**Fig. 1:**
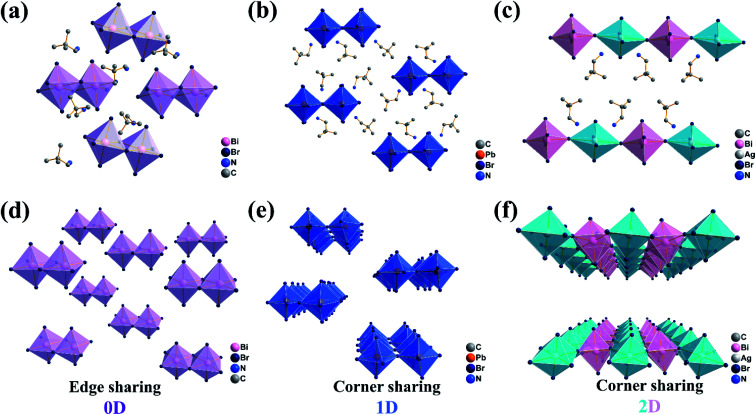
Basic structures of (DPA)_4_Bi_2_Br_10_ (a), (DPA)_5_Pb_2_Br_9_ (b) and (DPA)_4_AgBiBr_8_ (c) at low temperature. The dimension diagram of inorganic skeletons: (DPA)_4_Bi_2_Br_10_ (d), (DPA)_5_Pb_2_Br_9_ (e) and (DPA)_4_AgBiBr_8_ (f). The hydrogen atoms are omitted for clarity.

The crystal structures of (DPA)_4_Bi_2_Br_10_, (DPA)_5_Pb_2_Br_9_ and (DPA)_4_AgBiBr_8_ were determined by single crystal X-ray diffraction at low temperature. The structure of (DPA)_4_Bi_2_Br_10_ is characterized by structural analysis and crystallizes in the *P*1̄ (no. 2) space group of a triclinic system (Table S1, ESI†). It adopts zero-dimensional coordination packing, in which octahedrons are connected by edge sharing, and the N in the cation is oriented towards Br in the adjacent octahedron (Fig. 1a). Subsequently, the lead-replacement (DPA)_5_Pb_2_Br_9_ located in *P*1̄ (Table S1, ESI†) was successfully constructed. Unlike the traditional 1D face-sharing PbBr-based perovskites, (DPA)_5_Pb_2_Br_9_ adopts a unique double column corner-sharing connection to form a 1D chain. Due to the H-bond interaction, DPA cations in (DPA)_5_Pb_2_Br_9_ are interspersed orderly by N atoms facing the adjacent inorganic skeletons (Fig. 1b). Then two-dimensional (DPA)_4_AgBiBr_8_ was successfully assembled by a lead-free Ag/BiBr scheme, which also crystallizes in *P*1̄ (Table S1, ESI†). It is a corner sharing Ruddlesden–Popper perovskite structure. The N atoms in the upper and lower layers face the inorganic octahedron, showing an upward and downward posture respectively (Fig. 1c). In order to better present the structural dimension in the three compounds, an inorganic skeleton stacking as shown in Fig. 1d–f is drawn. The achievement is helpful to design and regulate the structural transformation with expected physicochemical features. All the structural information including hydrogen bonds, bond lengths, bond angles and hydrogen-bond geometry in the three compounds are listed in Fig. S1 and Tables S2–S7.†

### Analysis of phase transition behaviors

**Fig. 2:**
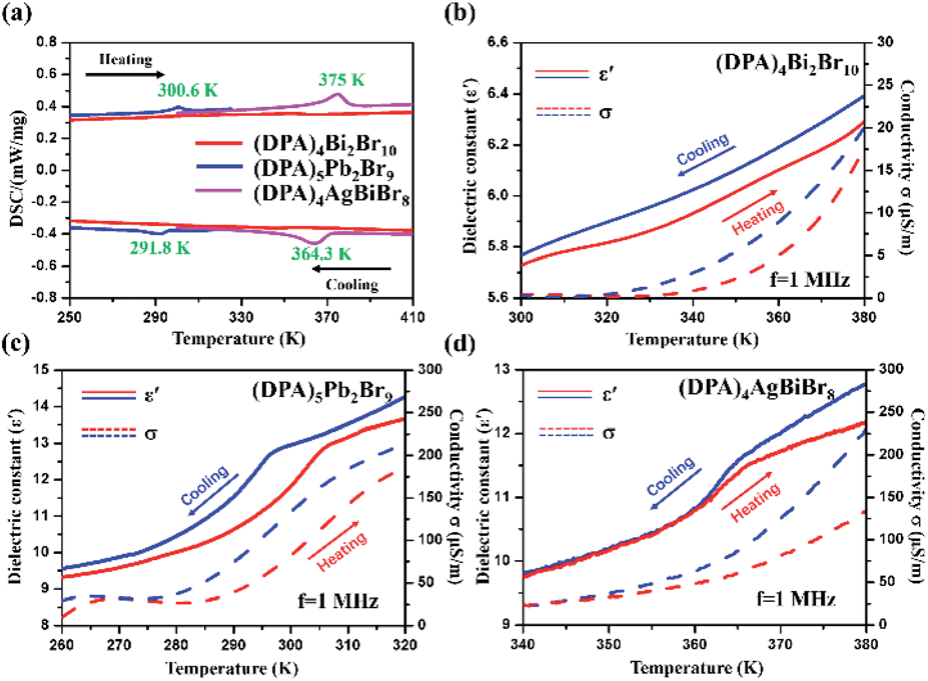
(a) DSC of (DPA)_4_Bi_2_Br_10_ (red), (DPA)_5_Pb_2_Br_9_ (blue) and (DPA)_4_AgBiBr_8_ (pink). Dielectric constant *ε*′ and conductivity *σ* of (DPA)_4_Bi_2_Br_10_ (b), (DPA)_5_Pb_2_Br_9_ (c) and (DPA)_4_AgBiBr_8_ (d) as a function of temperature.

Due to their potential application in temperature sensors and solid-to-solid phase transition substances, phase-transition features in the hybrids have attracted much attention.^46–48^ Therefore, various design strategies and construction schemes have been implemented in order to make new progress and improvement. The structural order feature provides a fixed stacking arrangement for the inorganic skeleton, so the charge-balance cations are also arranged regularly in the gap of the inorganic skeleton, and sufficient space is provided to realize possible thermal movement. Then the order–disorder structural phase transition is triggered, and a series of physical and chemical characteristics are induced. So, differential scanning calorimetry (DSC) and temperature-dependent dielectric measurements were carried out to prove the occurrence of phase transition. As speculated, (DPA)_5_Pb_2_Br_9_ and (DPA)_4_AgBiBr_8_ exhibit phase transition behavior at 300.6 K and 375 K in Fig. 2a, respectively. However, due to the different intermolecular forces, (DPA)_4_Bi_2_Br_10_ is not a phase transition one. And the temperature-dependent dielectric constant *ε*′ was obtained. The corresponding conductivity was calculated by using the formula *ε*′′ = *ε*′ tan *θ* and *σ*a.c. = *ωε*′′*ε*_0_, where *ε*_0_ is the permittivity of vacuum. The curves of the dielectric constant of (DPA)_4_Bi_2_Br_10_ (Fig. 2b) are nearly linear, and the dielectric constants for (DPA)_5_Pb_2_Br_9_ (Fig. 2c) and (DPA)_4_AgBiBr_8_ (Fig. 2d) are abnormal with the temperature heating/cooling. This is consistent with DSC analysis.

### Variable-temperature crystal structure analysis

**Fig. 3:**
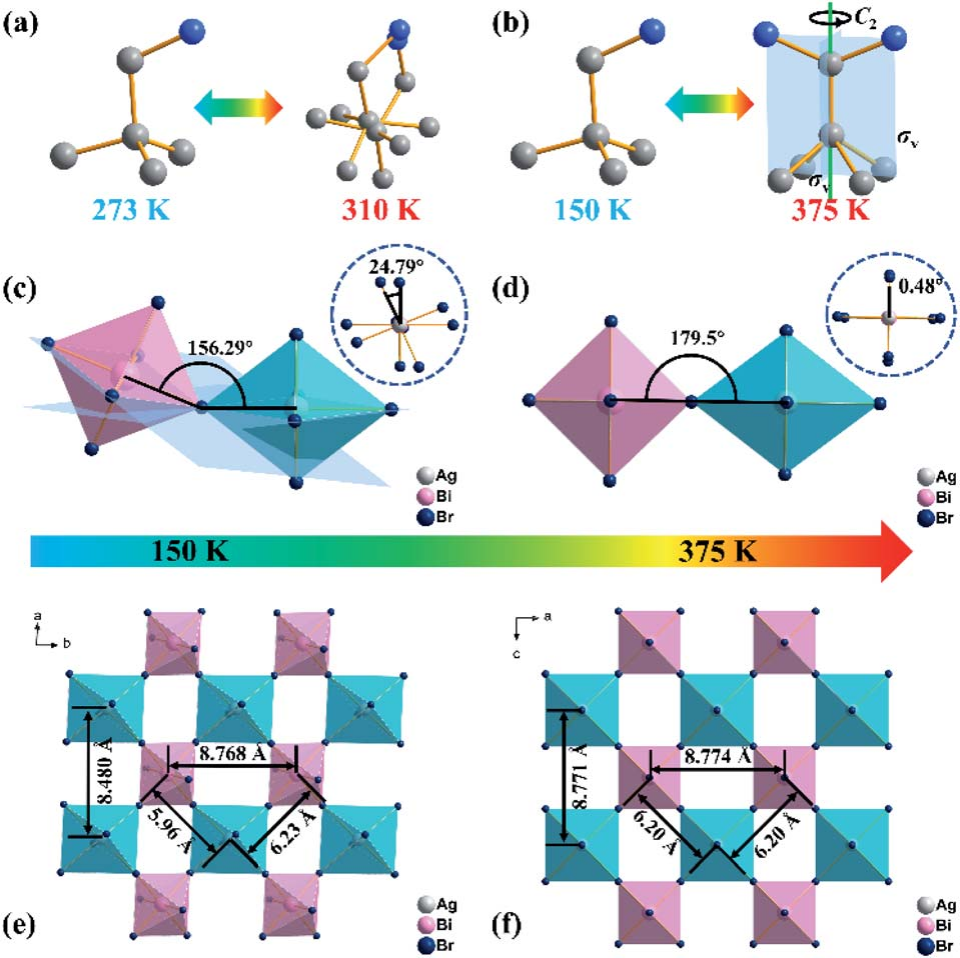
Movement of the cation in (DPA)_5_Pb_2_Br_9_ (a) and (DPA)_4_AgBiBr_8_ (b) in the low temperature and high temperature phase. Frontal and lateral (inset) torsion angles of a Bi-based octahedron with a Ag-based octahedron as the reference in (DPA)_4_AgBiBr_8_ in the low temperature (c) and high temperature phase (d). Distance between metal atoms of (DPA)_4_AgBiBr_8_ in the low temperature (e) and high temperature phase (f). The hydrogen atoms are omitted for clarity.

For solid-to-solid phase transition compounds (DPA)_5_Pb_2_Br_9_ and (DPA)_4_AgBiBr_8_, it is necessary to comprehend the relationship between the structures and physical properties. Therefore, single crystal X-ray diffraction of (DPA)_5_Pb_2_Br_9_ and (DPA)_4_AgBiBr_8_ in the high temperature phase was performed to determine the structures after phase transition. The space group of (DPA)_5_Pb_2_Br_9_ is also *P*1̄, and the bond lengths/angles of the inorganic skeleton change slightly (Tables S3 and S8, ESI†) at 310 K. Besides, as shown in Fig. 3a and S2a of the ESI,† half of the cations in (DPA)_5_Pb_2_Br_9_ undergo order–disorder transition in the high temperature phase, which is the main contributor for the phase transition.

In contrast, the space group of (DPA)_4_AgBiBr_8_ changes from *P*1̄ to *Cmmm* (no. 65), which can be classified as a ferroelastic phase transition with an Aizu notation of *mmmF*1̄. At 375 K, the DPA cation in (DPA)_4_AgBiBr_8_ is located at a special symmetry site of 2*mm* and undergoes molecular thermal vibration, which leads to its multi-oriented disordered state similar to the state of rotational motion (Fig. 3b and S2b, ESI†). In addition, the inorganic skeleton also changes significantly, and the frontal (156.29°) and lateral (24.79°, inset) torsion angles of the Bi-based octahedron with a Ag-based octahedron as the reference at 150 K (Fig. 3c) change to 179.5° and 0.48° at 375 K (Fig. 3d), indicating that the structure gradually changes from the distorted form to an inorganic perovskite-like structure. The change of the inorganic part including torsion of the octahedron and the shift of metal atoms is also observed from Fig. 3e and f, and the conclusion is consistent with the results discussed above. As another piece of evidence, variable-temperature powder X-ray diffraction of (DPA)_4_AgBiBr_8_ changes significantly (Fig. S3, ESI†), indicating that the phase transition occurred near 375 K.

### Variable-temperature solid-state NMR analysis

**Fig. 4:**
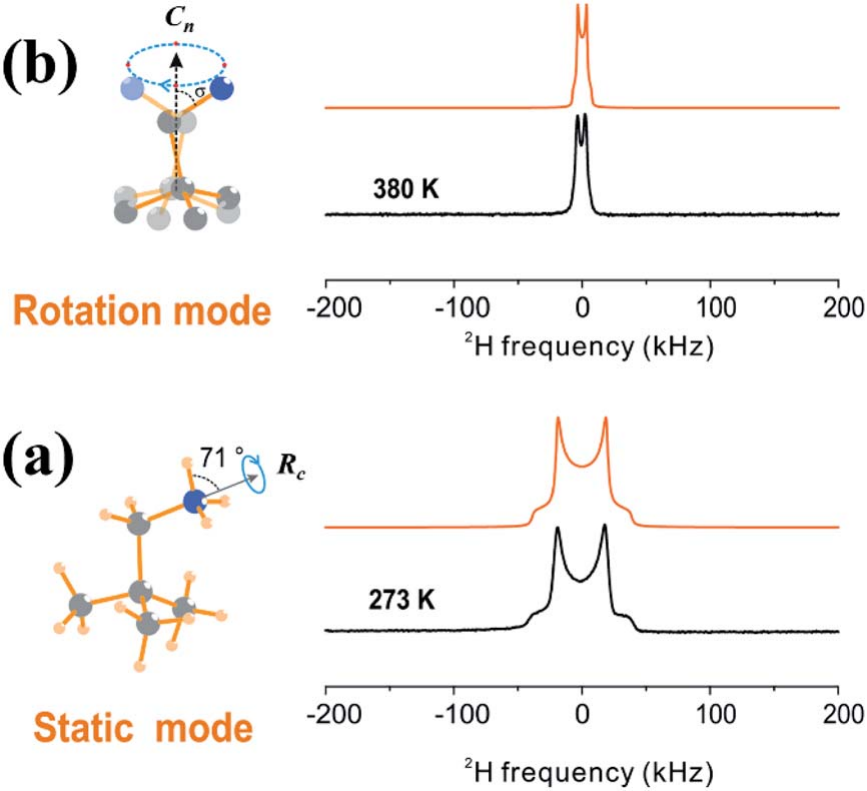
Experimental (black) and simulated (orange) ^2^H spectra of (DPA-d_3_)_4_AgBiBr_8_. This sample was synthesized by using N-deuterated DPA. The experimental spectra were acquired at 273 K (a) and 380 K (b). The simulated ^2^H spectra were obtained based on the motion model on the left *via* Weblab (https://weblab2.mpip-mainz.mpg.de/weblab66/weblab.html).

In order to prove the rotational motion of cations after phase transition, variable-temperature solid-state NMR was performed (solid-state NMR measurements, ESI†). Fig. 4 shows the experimental and simulated ^2^H NMR spectra of DPA cations at different temperatures. It can be observed that both the experimental spectra acquired at 273 K and 380 K exhibit axially symmetric powder Pake patterns, indicating that the DPA cations undergo some restricted reorientation processes. By simulating the spectra, we have obtained detailed information on the reorientation processes.

Fig. 4a shows the experimental and simulated ^2^H NMR spectra of DPA cations at 273 K. The distance between the two singularities, *ν*_QS_ is about 40 kHz. As discussed in the previous work on CH_3_ND_3_PbI_3_ (ref. 49) and CH_3_ND_3_NO_3_,^50^ such a value for *ν*_QS_ indicates that the ND_3_ group of the DPA cation performs rapid internal rotational motion about the axis of the C–N bond (*R*_C_ axis) with a rotation angle of about 71° and the whole molecular skeleton remains stationary (see Static mode in Fig. 4a). The spectrum simulated based on this motion model fits well with the experimental one.

When the temperature is just above the phase transition temperature (*e.g.*, 380 K), the Pake pattern exhibits a sudden narrowing and the *ν*_QS_ is motionally averaged to about 6 kHz, indicating that DPA cations undergo an overall cation reorientation (Fig. 4b). We introduce a motion model in which all the DPA cations perform a 4-site jump pattern along the *C*_*n*_ axis (close to the C–C axis, see rotation mode). The rotation angle, namely the included angle between the C–N bond (*R*_C_ axis) and *C*_*n*_ axis, is represented as *σ*. In addition, the internal rotational motion about the C–N bond (the *C*_2_ axis) also remains activated. Deuterium spectrum simulation is implemented based on this motion model and the result found that the spectrum simulated by using *σ* = 61.5° agrees very well with the experimental spectrum acquired at 380 K. It is worth noting that the bond angle of C–C–N is about 67°, very close to the rotation angle *σ*, indicating that the *C*_*n*_ axis is almost parallel to the C–C bond adjacent to the C–N bond in the DPA cation.

In general, combined with variable-temperature single crystal X-ray diffraction and variable-temperature solid-state NMR, we conclude that the phase transition of (DPA)_4_AgBiBr_8_ is due to the rotation of organic cations and the torsion of an octahedron in an inorganic skeleton.

### Ferroelasticity

**Fig. 5:**
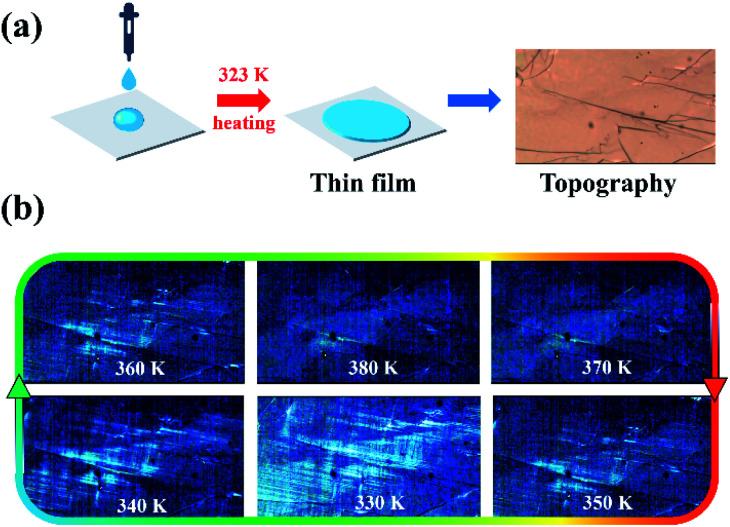
(a) Preparation process and topography of a thin film. (b) Evolution of ferroelastic domains of (DPA)_4_AgBiBr_8_ before and after phase-transition temperature (the contrast of the diagrams has been adjusted to observe the domain structures more clearly).

Based on the Aizu notation of *mmmF*1̄, (DPA)_4_AgBiBr_8_ can be classified as a typical ferroelastic. And the ferroelastic domains can be easily observed by using a variable-temperature polarizing microscope. Compared with inorganic materials, organic–inorganic hybrid ones can be characterized by both the bulk and thin films. Of course, the observation of thin films is the best choice to show clearer domain characteristics. Here, the thin film was prepared by dropping 20 μL of solution containing HBr acid (AR ≥ 40%, 500 μL) and (DPA)_4_AgBiBr_8_ (25 mg) on the ozone treated (20 minutes) ITO glass and then heating at 323 K for 45 minutes, as shown in Fig. 5a. By using a polarizing microscope at 330 K, the ferroelastic domain structures can be clearly observed (Fig. 5b). With the continuous increase of temperature from 330 K to 380 K, the domain structures gradually disappear near 375 K, suggesting the presence of the paraferroelastic phase in higher temperature, and then they gradually recover by cooling to the ferroelastic phase at 350 K. The disappearance and recovery of domain structures in the heating/cooling circles are completely consistent with the DSC, dielectric and temperature-dependence single structures.

The spontaneous strain tensor can be calculated by using the following matrix^51–53^ (1) according to its Aizu notation of *mmmF*1̄ from the high-symmetry phase (orthorhombic) to the low-symmetry phase (triclinic):1
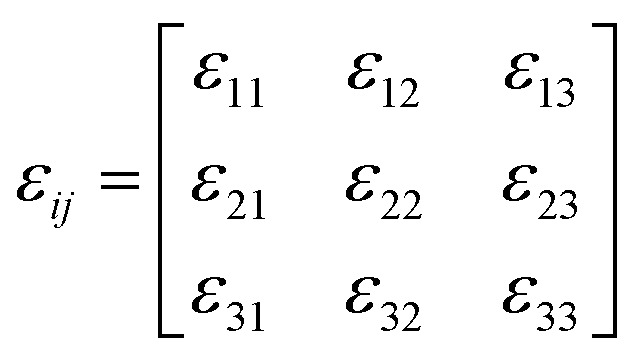


The formula corresponding to the elements in this matrix can be obtained in the ESI,† by substituting unit cell parameters at 150 K and 375 K in formula (1) and (2). In this way, a reasonable calculation is successfully completed, in which the total spontaneous strain *ε*_ss_ is 0.15593.2
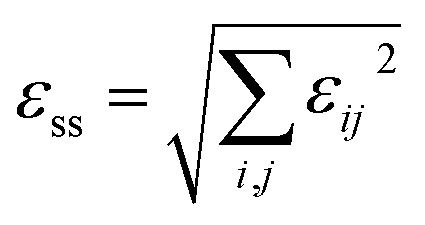


In addition, the domain transformation Video S1† was recorded, showing the readers a beautiful reversible ferroelastic transition process. This is very helpful to analyze and explore the domain structure and the micro mechanism.

### Semiconducting behavior

**Fig. 6:**
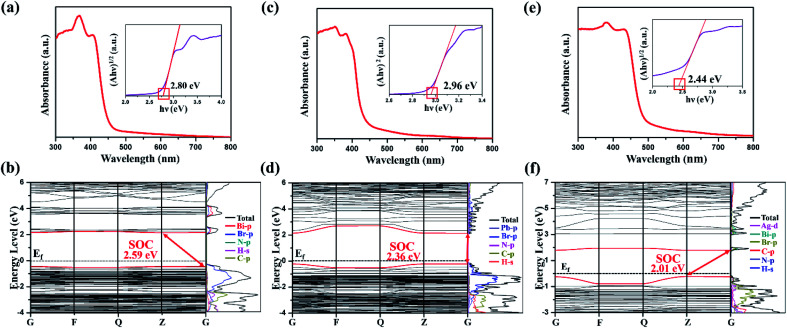
The UV-vis absorption spectra, corresponding Tauc plots, and calculated energy band structure of (DPA)_4_Bi_2_Br_10_ (a) and (b), (DPA)_5_Pb_2_Br_9_ (c) and (d) and (DPA)_4_AgBiBr_8_ (e) and (f) with spin–orbit coupling (SOC), and corresponding partial density of states (PDOS).

The UV-vis absorption and density functional theory (DFT) calculations of (DPA)_4_Bi_2_Br_10_, (DPA)_5_Pb_2_Br_9_ and (DPA)_4_AgBiBr_8_ were implemented to consider and discuss the change of their band gaps in Fig. 6. As shown in the Tauc plot, (DPA)_4_AgBiBr_8_ exhibits an indirect band gap of 2.44 eV, which is less than that of (DPA)_4_Bi_2_Br_10_ (indirect, 2.80 eV) and (DPA)_5_Pb_2_Br_9_ (direct, 2.96 eV). As a Ruddlesden–Popper type double perovskite, the band gap of (DPA)_4_AgBiBr_8_ is close to that of other reported similar AgBiBr-based compounds (Table S9, ESI†), such as 2.41 eV in (PA)_4_AgBiBr_8_ (PA = propylammonium),^32^ 2.43 eV in (BDA)_4_AgBiBr_8_ (BDA = butane-1,4-diammonium)^32^ and 2.45 eV in (OcA)_4_AgBiBr_8_ (OcA = octylammonium),^32^ which is lower than that of BA-based (BA = butylammonium) double perovskite^54,55^ and higher than that of Cs^+^ double perovskite.^32^ By comparing with AgBiI-based ones, the band gap of AgBiBr-based double perovskite is significantly higher (Table S9, ESI†).^30,56^ Therefore, it can be found that the influence of the inorganic part on the band gap is greater than that of the organic part.

With spin-orbital coupling (SOC), the band gap, the valence band maximum (VBM) and the conduction band minimum (CBM) in the three compounds are predicted in Fig. 6b, d and f. In addition, the energy band structure of (DPA)_4_AgBiBr_8_ without SOC was obtained (Fig. S4, ESI†).The differences between SOC and non-SOC are mainly derived from a split-off conduction band within Bi-6p states according to the partial density of states, and a precise prediction implies the necessity of SOC in Bi-based and Pb-based organic–inorganic perovskite.^45,57–59^ For these three compounds, the valence band maximum (VBM) and conduction band minimum (CBM) are mainly contributed by the inorganic part. Besides, in (DPA)_4_Bi_2_Br_10_ (Fig. 6b), (DPA)_5_Pb_2_Br_9_ (Fig. 6d) or (DPA)_4_AgBiBr_8_ (Fig. 6f), the organic part, and the H-s, N-p and C-p states overlap widely, indicating a strong interaction.

## Conclusions

In this work, three target compounds were synthesized through the metal substitution strategy as shown in Scheme 1. In particular, an AgBi-based hybrid double perovskite was successfully constructed. The (DPA)_5_Pb_2_Br_9_ and (DPA)_4_AgBiBr_8_ show solid-to-solid phase transition. It is worth mentioning that (DPA)_4_AgBiBr_8_ is a high temperature ferroelastic material classified as *mmmF*1̄, and its phase transition comes from the rotational cation motion and the torsion of the octahedron in the inorganic skeleton. Theconstruction of (DPA)_4_AgBiBr_8_ broadens the potential applications of two-dimensional double perovskite. At the same time, (DPA)_4_AgBiBr_8_ also provides power for the explosion of new ferroelastics or hybrid double perovskites.

## Experimental

### Synthesis

All the reagents and solvents mentioned in this work were purchased from commercial suppliers and were not further purified.

### (2,2-Dimethylpropan-1-aminium)_4_Bi_2_Br_10_ (DPA)_4_Bi_2_Br_10_

Stoichiometric amounts of 2,2-dimethylpropan-1-amine (0.2 mmol) and bismuth bromide (0.2 mmol) were added into a beaker. After this, hydrobromic acid (AR ≥ 40%, 20 mL) was added into the beaker. A colorless prism crystal of (DPA)_4_Bi_2_Br_10_ was obtained for single-crystal X-ray diffraction study through slow evaporation of the mixed solution at room temperature after several days.

### (2,2-Dimethylpropan-1-aminium)_5_Pb_2_Br_9_ (DPA)_5_Pb_2_Br_9_

Except that bismuth bromide was replaced by lead bromide (0.2 mmol), the other steps of synthesizing (DPA)_5_Pb_2_Br_9_ are the same as the synthesis of (DPA)_4_Bi_2_Br_10_. Eventually, yellow block crystals of (DPA)_5_Pb_2_Br_9_ were obtained successfully.

### (2,2-Dimethylpropan-1-aminium)_4_AgBiBr_8_ (DPA)_4_AgBiBr_8_

A round-bottom flask containing a turbid liquid of Ag_2_O (0.5 mmol), Bi_2_O_3_ (0.5 mmol), 2,2-dimethylpropan-1-amine (10 mmol) and HBr acid (AR ≥ 40%, 10 mL) was heated at 373 K to obtain a pellucid yellow solution. Subsequently, the yellow block crystals of (DPA)_4_AgBiBr_8_ for single-crystal XRD were obtained after slow cooling. Powder X-ray diffraction (PXRD) for verifying the purity and thermogravimetric analysis (TGA) for indicating the stability of (DPA)_4_Bi_2_Br_10_, (DPA)_5_Pb_2_Br_9_ and (DPA)_4_AgBiBr_8_ are shown in Fig. S5 and S6 of the ESI†.

## Author contributions

C.-Y. S. conceived and conducted the experiments, analyzed the data and wrote the paper. Z.-X. Z. carried out the observation of ferroelastic domains. Y. W. and M. C. carried out the dielectric characterization studies. W.-C. Q. and Y.-F. Y. carried out solid-state NMR. P.-Z. H. assisted in data analysis. D.-W. F. and Y. Z. guided and supervised this work.

## Conflicts of interest

The authors declare no conflict of interest.

## Supplementary Material

SC-013-D1SC07045B-s001

SC-013-D1SC07045B-s002

SC-013-D1SC07045B-s003
