# Supplementary material for: The construction of a two-dimensional organic–inorganic hybrid double perovskite ferroelastic with a high Tc and narrow band gap
Source: Chem Sci. 2022 Mar 30;13(17):4794–800. doi: 10.1039/d1sc07045b (PMC9067571; doi:10.1039/d1sc07045b)
Supplement: SC-013-D1SC07045B-s001 [file SC-013-D1SC07045B-s001.pdf]

Electronic Supplementary Material (ESI) for Chemical Science.
This journal is © The Royal Society of Chemistry 2022

Supplementary information

Table of contents

Experimental Measurement Methods S2

Physical measurements. S2

DSC Measurements. S2

Single-Crystal X-ray Crystallography. S2

Solid-state NMR measurements. S2

The equations for calculation of the spontaneous strain. S3

Density Functional Theory Calculations. S3

Figure S1. Hydrogen bonds of $(DPA)_4Bi_2Br_{10}$ (a), $(DPA)_5Pb_2Br_9$ (b) and $(DPA)_4AgBiBr_8$ (c). S3

Figure S2. Packing diagram of compound S4

Figure S3. (a) Two-dimensional powder diffraction pattern of $(DPA)_4AgBiBr_8$ before (350 K) and after (390 K) phase transition temperature. (b) Variable-temperature powder X-ray diffraction patterns of $(DPA)_4AgBiBr_8$ from 350 K to 390 K. S4

Figure S4. Calculated energy band structure of $(DPA)_4AgBiBr_8$ without spin-orbit coupling (SOC), and corresponding partial density of states (PDOS). S5

Table S1. Crystal data and structure refinements for $(DPA)_4Bi_2Br_{10}$, $(DPA)_5Pb_2Br_9$ and $(DPA)_4AgBiBr_8$. S5

Table S2. Selected bond lengths [Å] and bond angles [°] for $(DPA)_4Bi_2Br_{10}$ at 200 K. S6

Table S3. Selected bond lengths [Å] and bond angles [°] for $(DPA)_5Pb_2Br_9$ at 273 and 310 K. S7

Table S4. Selected bond lengths [Å] and bond angles [°] for $(DPA)_4AgBiBr_8$ at 150 and 375 K. S8

Table S5. Hydrogen-bond geometry (Å, °) for $(DPA)_4Bi_2Br_{10}$ at 200 K. S9

Table S6. Hydrogen-bond geometry (Å, °) for $(DPA)_5Pb_2Br_9$ at 273 K. S10

Table S7. Hydrogen-bond geometry (Å, °) for $(DPA)_4AgBiBr_8$ at 150 K. S11

Table S8. Crystal data and structure refinements for $(DPA)_5Pb_2Br_9$ and $(DPA)_4AgBiBr_8$ at high temperature phase. S11

Table S9. The reported space group and band gap of Ruddlesden−Popper AgBiBr- and AgBiI-based materials (n=1). S12

Appendix S1. Acronyms arranged in top-down order in Table S9. .... S12

Reference: S12

Experimental Measurement Methods

Physical measurements. Differential scanning calorimetry (DSC), variable-temperature single-crystal X-ray diffraction, powder X-ray diffraction (PXRD) measurements, dielectric measurements, ultraviolet-vis spectral measurements and electronic structure calculations were described in Supporting Information.

DSC Measurements.

DSC measurement of **$(DPA)_4Bi_2Br_{10}$**, **$(DPA)_5Pb_2Br_9$** and **$(DPA)_4AgBiBr_8$** was carried out in the temperature range from 173 to 453 K at a rate of 20 K min^{-1} using a PerkinElmer Diamond DSC instrument.

Single-Crystal X-ray Crystallography.

Variable-temperature single-crystal XRD data of **$(DPA)_4Bi_2Br_{10}$**, **$(DPA)_5Pb_2Br_9$** and **$(DPA)_4AgBiBr_8$** were collected using a Bruker APEX-II CCD with Mo Kα radiation (λ = 0.71073 Å) at different temperature, whose processing was disposed by the APEX3. Variable-temperature crystal structures were solved using a direct method and subsequent continuous Fourier synthesis. Subsequently, these crystals were refined by full-matrix leastsquares methods based on F2 using the SHELXTL software package. Eventually, in addition to the asymmetric units and packing shown in main body using DIAMOND software, other relevant crystallographic data and structure refinement are listed in **Table S1** and **S2** in supporting information.

Solid-state NMR measurements.

Like the synthesis of $(DPA)_4AgBiBr_8$, in the synthesis process, HBr acid (AR≥40%, 10 ml) was replaced with DBr (48% w/w in D_2O, 99+%, 10 g). Eventually, the yellow block crystals of $(DPA\text{-}d_3)_4AgBiBr_8$ for single-crystal XRD were obtained after slow cooling.

Wide-line ^2H NMR studies were performed by using a Bruker Avance III 300 spectrometer operating at 46.07 MHz. The ^2H spectra were recorded by using a Bruker two-channel static polarization enhancement (PE) probe with a homemade 2.5 mm coil. The spectra were acquired by using a solid echo pulse sequence with a pulse width of 2 μs at a radio-frequency (RF) amplitude of $\gamma B_1/2\pi$ = 125 kHz. The refocusing delay τ

was approximately 30 μs. The ^{2}H spectra were simulated by using the weblab software (https://weblab2.mpip-mainz.mpg.de/weblab66/weblab.html).

The equations for calculation of the spontaneous strain.

For $mmm\mathrm{F}\bar{1}$, the spontaneous strain tensor can be calculated by the following matrix:

$$\varepsilon_{ij}=\begin{bmatrix} \frac{a_{RTP}}{a_{HTP}}\sin\gamma_{RTP}-1 & \frac{1}{2}\left(\frac{a_{RTP}}{a_{HTP}}\cos\gamma_{RTP}\right) & \frac{1}{2}\left(-\frac{c_{RTP}\sin\alpha_{RTP}\cos(180°-\beta_{RTP})}{c'_{HTP}}\right) \\ \frac{1}{2}\left(\frac{a_{RTP}}{a_{HTP}}\cos\gamma_{RTP}\right) & \frac{b_{RTP}}{b'_{HTP}}-1 & \frac{1}{2}\left(\frac{c_{RTP}\cos\alpha_{RTP}}{c'_{HTP}}\right) \\ \frac{1}{2}\left(-\frac{c_{RTP}\sin\alpha_{RTP}\cos(180°-\beta_{RTP})}{c'_{HTP}}\right) & \frac{1}{2}\left(\frac{c_{RTP}\cos\alpha_{RTP}}{c'_{HTP}}\right) & \frac{c_{RTP}\sin\alpha_{RTP}\sin(180°-\beta_{RTP})}{c'_{HTP}}-1 \end{bmatrix}$$

The total spontaneous strain ε_{ss} can be calculated by $\varepsilon_{ss}=\sqrt{\sum_{ij}\varepsilon_{ij}^2}$.

Density Functional Theory Calculations.

Density functional theory (DFT) were performed with the Vienna Ab Initio Simulation Package (VASP)[1]. The Perdew−Burke−Ernzerhof(PBE)[2] functional and frozen-core all electron projector augmented wave (PAW)[3] model was employed. The cutoff energy was set to 500 eV. And the convergence criteria for the residual force and energy on each atom during structure relaxation were set to 0.01 eV/Å and 10^{-5} eV, respectively.

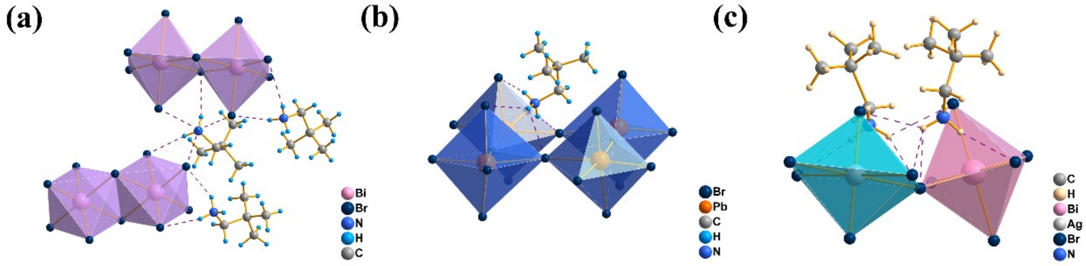

Figure S1. Hydrogen bonds of $\mathbf{(DPA)_4Bi_2Br_{10}}$ (a), $\mathbf{(DPA)_5Pb_2Br_9}$ (b) and $\mathbf{(DPA)_4AgBiBr_8}$ (c).

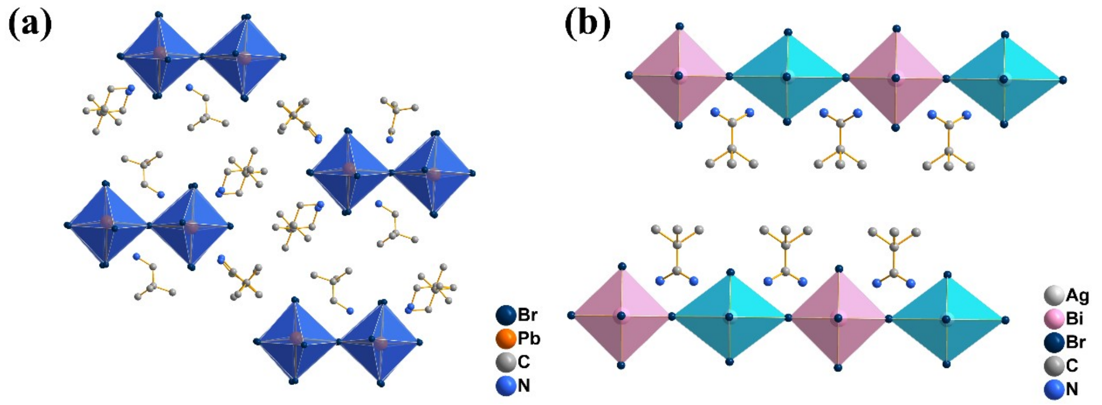

Figure S2. Packing diagram of compound **$(DPA)_5Pb_2Br_9$** (a) and **$(DPA)_4AgBiBr_8$** (b) at high temperature.

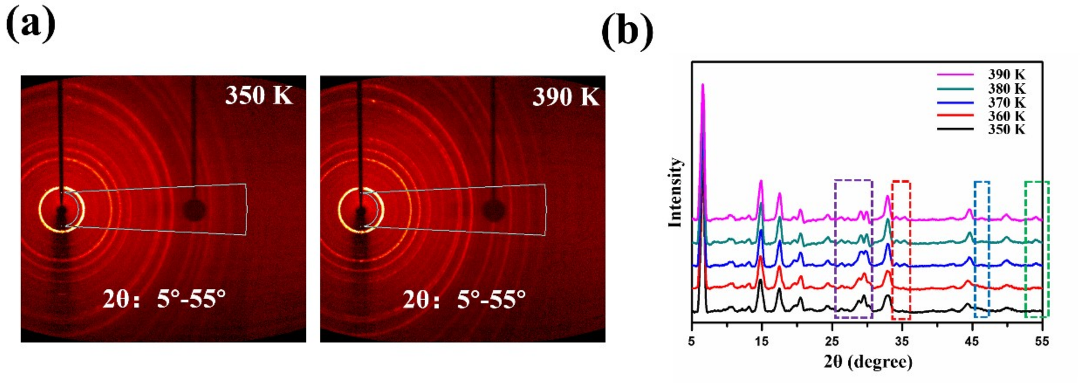

Figure S3. (a) Two-dimensional powder diffraction pattern of **$(DPA)_4AgBiBr_8$** before (350 K) and after (390 K) phase transition temperature. (b) Variable-temperature powder X-ray diffraction patterns of **$(DPA)_4AgBiBr_8$** from 350 K to 390 K.

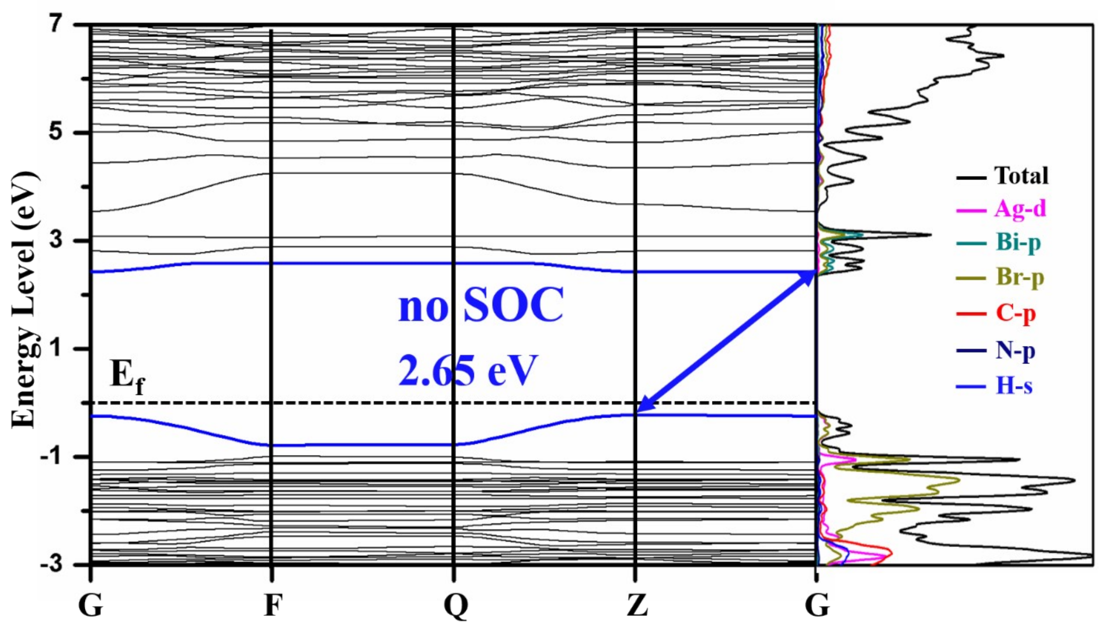

Figure S4. Calculated energy band structure of **$(DPA)_4AgBiBr_8$** without spin-orbit coupling (SOC), and corresponding partial density of states (PDOS).

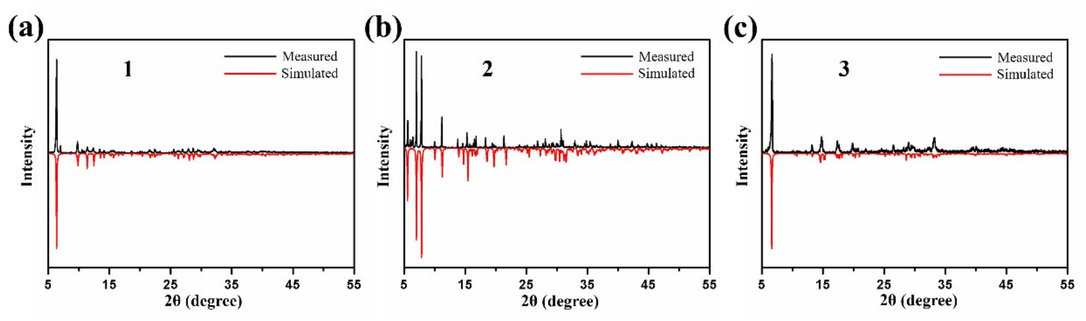

Figure S5. Measured and simulated powder X-ray diffraction patterns of **$(DPA)_4Bi_2Br_{10}$**, **$(DPA)_5Pb_2Br_9$** and **$(DPA)_4AgBiBr_8$**.

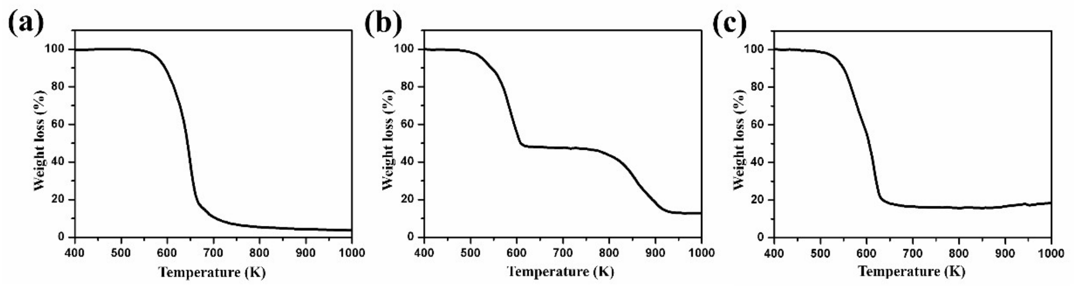

Figure S6. TGA curve of **$(DPA)_4Bi_2Br_{10}$**, **$(DPA)_5Pb_2Br_9$** and **$(DPA)_4AgBiBr_8$**.

Table S1. Crystal data and structure refinements for **$(DPA)_4Bi_2Br_{10}$, $(DPA)_5Pb_2Br_9$ and $(DPA)_4AgBiBr_8$.**

	**$(DPA)_4Bi_2Br_{10}$** 200 K	**$(DPA)_5Pb_2Br_9$** 273 K	**$(DPA)_4AgBiBr_8$** 150 K
Empirical formula	$C_{20}H_{56}Bi_2Br_{10}N_4$	$C_{25}H_{70}Br_9N_5Pb_2$	$C_{20}H_{56}AgBiBr_8N_4$
Formula weight	1569.74	1574.43	1308.74
Crystal system	Triclinic	Triclinic	Triclinic
Space group	$P\bar{1}$	$P\bar{1}$	$P\bar{1}$
a (Å)	12.4378(19)	11.975 (2)	8.4797(6)
b (Å)	13.2166(19)	13.366 (2)	8.7683(6)
c (Å)	14.021(3)	17.472 (3)	13.9411(9)
α (°)	97.835(4)	72.618 (5)	73.253(3)
β (°)	93.139(5)	70.213 (4)	88.908(3)
γ (°)	90.289(4)	77.258 (4)	87.415(3)
Volume(Å^3)	2279.7(6)	2488.9 (8)	991.56(12)
Z	2	2	1
Radiation type	MoK\a	MoK\a	MoK\a
Absorption correction	Multi-scan	Multi-scan	Multi-scan
D_{calc} /g cm^{-3}	2.287	2.101	2.192

F (000)	1440	1468	614
GOF	1.022	1.052	1.095
$R_1[I > 2\sigma(I)]$	0.0492	0.0658	0.1097
$wR2[I > 2\sigma(I)]$	0.1202	0.2002	0.3188

Table S2. Selected bond lengths [Å] and bond angles [°] for **$(DPA)_4Bi_2Br_{10}$** at 200 K.

Temperature	bond lengths [Å]		bond angles [°]	
200 K	Bi01—Br0B	2.7076 (12)	Br0B—Bi01—Br0A	93.49 (4)
	Bi01—Br0A	2.7782 (10)	Br0B—Bi01—Br06	92.43 (3)
	Bi01—Br06	2.7841 (9)	Br0A—Bi01—Br06	88.32 (3)
	Bi01—Br05	2.9184 (10)	Br0B—Bi01—Br05	92.84 (3)
	Bi01—Br07	3.0015 (10)	Br0A—Bi01—Br05	92.31 (3)
	Bi01—$Br07^{i}$	3.0754 (12)	Br06—Bi01—Br05	174.64 (3)
	Bi02—Br0C	2.6995 (13)	Br0B—Bi01—Br07	88.79 (3)
	Bi02—Br04	2.7749 (10)	Br0A—Bi01—Br07	177.32 (3)
	Bi02—Br09	2.7862 (10)	Br06—Bi01—Br07	90.17 (3)
	Bi02—Br03	2.9298 (10)	Br05—Bi01—Br07	88.99 (3)
	Bi02—Br08	3.0014 (10)	Br0B—Bi01—$Br07^{i}$	175.47 (3)
	Bi02—$Br08^{ii}$	3.0951 (12)	Br0A—Bi01—$Br07^{i}$	90.91 (3)
			Br06—Bi01—$Br07^{i}$	88.78 (3)
			Br05—Bi01—$Br07^{i}$	85.88 (3)
			Br07—Bi01—$Br07^{i}$	86.84 (3)
			Br0C—Bi02—Br04	92.77 (3)
			Br0C—Bi02—Br09	94.29 (4)
			Br04—Bi02—Br09	87.72 (3)
			Br0C—Bi02—Br03	91.93 (3)
			Br04—Bi02—Br03	175.27 (3)
			Br09—Bi02—Br03	92.48 (3)
			Br0C—Bi02—Br08	89.79 (3)
			Br04—Bi02—Br08	88.60 (3)
			Br09—Bi02—Br08	174.64 (3)
			Br03—Bi02—Br08	90.87 (3)
			Br0C—Bi02—$Br08^{ii}$	174.98 (3)
			Br04—Bi02—$Br08^{ii}$	88.21 (3)
			Br09—Bi02—$Br08^{ii}$	90.67 (3)
			Br03—Bi02—$Br08^{ii}$	87.06 (3)
			Br08—Bi02—$Br08^{ii}$	85.31 (3)
			Bi01—Br07—$Bi01^{i}$	93.16 (3)
			Bi02—Br08—$Bi02^{ii}$	94.69 (3)

Symmetry codes: (i) $-x$, $-y$+1, $-z$+1; (ii) $-x$+1, $-y$+2, $-z$+1. (200 K)

Table S3. Selected bond lengths [Å] and bond angles [°] for **$(DPA)_5Pb_2Br_9$** at 273 and 310 K.

Temperature	bond lengths [Å]		bond angles [°]	
273 K	Br1—Pb1	3.1158 (13)	Pb1—Br1—Pb2^{i}	162.60 (6)
	Br1—Pb2^{i}	3.1851 (14)	Pb1—Br5—Pb2	168.15 (5)
	Br2—Pb1	3.0801 (15)	Pb2—Br8—Pb1^{ii}	151.29 (4)
	Br3—Pb1	2.9570 (13)	Br4—Pb1—Br3	89.97 (4)
	Br4—Pb1	2.9458 (13)	Br4—Pb1—Br5	95.53 (4)
	Br5—Pb1	3.0082 (12)	Br3—Pb1—Br5	98.38 (4)
	Br5—Pb2	3.0278 (12)	Br4—Pb1—Br2	174.73 (4)
	Br6—Pb2	2.9032 (13)	Br3—Pb1—Br2	85.87 (4)
	Br7—Pb2	3.1475 (15)	Br5—Pb1—Br2	88.27 (4)
	Br8—Pb2	3.0773 (12)	Br4—Pb1—Br8^{iii}	85.37 (4)
	Br8—Pb1^{ii}	3.0918 (12)	Br3—Pb1—Br8^{iii}	90.98 (4)
	Br9—Pb2	2.8852 (15)	Br5—Pb1—Br8^{iii}	170.59 (3)
			Br2—Pb1—Br8^{iii}	91.47 (4)
			Br4—Pb1—Br1	92.79 (4)
			Br3—Pb1—Br1	175.45 (4)
			Br5—Pb1—Br1	84.96 (4)
			Br2—Pb1—Br1	91.18 (4)
			Br8^{iii}—Pb1—Br1	85.64 (4)
			Br9—Pb2—Br6	90.43 (4)
			Br9—Pb2—Br5	95.91 (4)
			Br6—Pb2—Br5	95.66 (4)
			Br9—Pb2—Br8	91.03 (4)
			Br6—Pb2—Br8	89.88 (4)
			Br5—Pb2—Br8	171.07 (3)
			Br9—Pb2—Br7	173.83 (4)
			Br6—Pb2—Br7	84.29 (4)
			Br5—Pb2—Br7	87.81 (4)
			Br8—Pb2—Br7	85.78 (4)
			Br9—Pb2—Br1^{i}	99.62 (4)
			Br6—Pb2—Br1^{i}	169.26 (4)
			Br5—Pb2—Br1^{i}	87.13 (4)
			Br8—Pb2—Br1^{i}	86.17 (4)
			Br7—Pb2—Br1^{i}	85.46 (4)
310 K	Br1—Pb2	3.0715 (12)	Pb2—Br1—Pb1^{i}	152.03 (5)
	Br1—Pb1^{i}	3.0862 (12)	Pb1—Br2—Pb2	168.88 (6)
	Br2—Pb1	3.0189 (12)	Pb1—Br6—Pb2^{ii}	164.86 (6)
	Br2—Pb2	3.0338 (12)	Br3—Pb1—Br4	90.34 (4)
	Br3—Pb1	2.9389 (13)	Br3—Pb1—Br2	98.68 (4)
	Br4—Pb1	2.9554 (14)	Br4—Pb1—Br2	95.44 (4)
	Br5—Pb2	2.8957 (13)	Br3—Pb1—Br8	85.54 (4)

Br6—Pb1	3.1315 (13)	Br4—Pb1—Br8	174.65 (4)
Br6—Pb2^{ii}	3.1833 (14)	Br2—Pb1—Br8	88.57 (4)
Br7—Pb2	2.9059 (15)	Br3—Pb1—Br1^{iii}	90.72 (4)
Br8—Pb1	3.0852 (15)	Br4—Pb1—Br1^{iii}	85.72 (4)
Br9—Pb2	3.1416 (16)	Br2—Pb1—Br1^{iii}	170.51 (4)
		Br8—Pb1—Br1^{iii}	90.91 (4)
		Br3—Pb1—Br6	174.77 (4)
		Br4—Pb1—Br6	92.63 (4)
		Br2—Pb1—Br6	85.33 (4)
		Br8—Pb1—Br6	91.22 (4)
		Br1^{iii}—Pb1—Br6	85.21 (4)
		Br5—Pb2—Br7	90.45 (4)
		Br5—Pb2—Br2	96.40 (4)
		Br7—Pb2—Br2	95.90 (4)
		Br5—Pb2—Br1	89.68 (4)
		Br7—Pb2—Br1	90.49 (4)
		Br2—Pb2—Br1	171.13 (4)
		Br5—Pb2—Br9	84.45 (5)
		Br7—Pb2—Br9	173.93 (4)
		Br2—Pb2—Br9	87.96 (4)
		Br1—Pb2—Br9	86.20 (4)
		Br5—Pb2—Br6^{ii}	170.20 (4)
		Br7—Pb2—Br6^{ii}	98.19 (4)
		Br2—Pb2—Br6^{ii}	87.35 (4)
		Br1—Pb2—Br6^{ii}	85.65 (4)
		Br9—Pb2—Br6^{ii}	86.64 (4)

Symmetry codes: (i) −*x*+1, −*y*+1, −*z*+1; (ii) *x*−1, *y*, *z*; (iii) *x*+1, *y*, *z*. (273 K)
Symmetry codes: (i) *x*−1, *y*, *z*; (ii) −*x*+1, −*y*+1, −*z*+1; (iii) *x*+1, *y*, *z*. (310 K)

Table S4. Selected bond lengths [Å] and bond angles [°] for **$(DPA)_4AgBiBr_8$** at 150 and 375 K.

Temperature	bond lengths [Å]		bond angles [°]	
150 K	Bi1—Br1^{i}	2.827 (2)	Br1^{i}—Bi1—Br1	180.0
	Bi1—Br1	2.827 (2)	Br1^{i}—Bi1—Br3	92.26 (7)
	Bi1—Br3	2.8347 (19)	Br1—Bi1—Br3	87.74 (7)
	Bi1—Br3^{i}	2.8348 (19)	Br1^{i}—Bi1—Br3^{i}	87.74 (7)
	Bi1—Br2^{i}	2.873 (2)	Br1—Bi1—Br3^{i}	92.26 (7)
	Bi1—Br2	2.873 (2)	Br3—Bi1—Br3^{i}	180.00 (8)
	Ag1—Br4^{ii}	2.547 (3)	Br1^{i}—Bi1—Br2^{i}	88.43 (11)

	Ag1—Br4	2.547 (3)	Br1—Bi1—Br2^{i}	91.57 (11)
			Br3—Bi1—Br2^{i}	91.07 (7)
			Br3^{i}—Bi1—Br2^{i}	88.93 (7)
			Br1^{i}—Bi1—Br2	91.57 (11)
			Br1—Bi1—Br2	88.43 (11)
			Br3—Bi1—Br2	88.93 (7)
			Br3^{i}—Bi1—Br2	91.07 (7)
			Br2^{i}—Bi1—Br2	180.0
			Br4^{ii}—Ag1—Br4	180.0
375 K	Ag1—Br1^{i}	2.538 (13)	Br1^{i}—Ag1—Br1	180.0
	Ag1—Br1	2.538 (13)	Br1^{i}—Ag1—Br3	90.000 (1)
	Ag1—Br3	3.335 (9)	Br1—Ag1—Br3	90.000 (1)
	Bi1—Br2^{ii}	2.816 (13)	Br2^{ii}—Bi1—Br2	180.0
	Bi1—Br2	2.816 (13)	Br2^{ii}—Bi1—Br3	90.000 (1)
	Bi1—Br3	2.868 (9)	Br2—Bi1—Br3	90.000 (1)
	Bi1—Br3^{ii}	2.868 (9)	Br2^{ii}—Bi1—Br3^{ii}	90.000 (1)
	Bi1—Br3^{iii}	2.868 (9)	Br2—Bi1—Br3^{ii}	90.000 (2)
	Bi1—Br3^{iv}	2.868 (9)	Br3—Bi1—Br3^{ii}	180.0
			Br2^{ii}—Bi1—Br3^{iii}	90.000 (3)
			Br2—Bi1—Br3^{iii}	90.000 (1)
			Br3—Bi1—Br3^{iii}	90.5 (6)
			Br3^{ii}—Bi1—Br3^{iii}	89.5 (5)
			Br2^{ii}—Bi1—Br3^{iv}	90.000 (1)
			Br2—Bi1—Br3^{iv}	90.000 (3)
			Br3—Bi1—Br3^{iv}	89.5 (5)
			Br3^{ii}—Bi1—Br3^{iv}	90.5 (6)
			Br3^{iii}—Bi1—Br3^{iv}	180.0
			Bi1—Br3—Ag1	179.5 (5)

Symmetry codes: (i) −*x*+1, −*y*+1, −*z*+1; (ii) −*x*+2, −*y*+2, −*z*+1; (iii) −*x*+1, −*y*+2, −*z*+1; (iv) *x*+1, *y*, *z*. (150 K)
Symmetry codes: (i) −*x*, −*y*+1, −*z*; (ii) −*x*+1, −*y*+1, −*z*+1; (iii) *x*, *y*, −*z*+1; (iv) −*x*+1, −*y*+1, *z*; (v) −*x*+1, *y*, −*z*+2; (vi) −*x*+1, *y*, *z*; (vii) *x*, *y*, −*z*+2; (viii) −*x*, *y*, *z*; (ix) −*x*, *y*, −*z*+1. (375 K)

Table S5. Hydrogen-bond geometry (Å, °) for **$(DPA)_4Bi_2Br_{10}$** at 200 K.

D—H···*A*	*D*—H	H···*A*	*D*···*A*	*D*—H···*A*
C9—H9*B*···Br08^{iii}	0.99	3.03	3.835 (14)	139
C00*N*—H00*P*···Br03	0.99	2.99	3.635 (15)	124
C00*N*—H00*O*···Br0*B*	0.99	3.01	3.982 (15)	167
C00*M*—H00*M*···Br07^{iv}	0.99	2.97	3.787 (14)	141
C3*H*—H3*H*2···Br0*C*	0.99	3.04	4.009 (14)	166
C3*H*—H3*H*1···Br05^{iv}	0.99	3.02	3.659 (14)	123

N00*G*—H00*L*···Br06^{iv}	0.91	2.60	3.423 (8)	150
N00*G*—H00*K*···Br05^{v}	0.91	2.74	3.488 (8)	140
N00*G*—H00*K*···Br04^{iii}	0.91	2.97	3.534 (8)	122
N00*G*—H00*J*···Br09^{iii}	0.91	2.61	3.485 (8)	163
N00*F*—H00*I*···Br0*A*	0.91	2.71	3.514 (8)	148
N00*F*—H00*H*···Br06	0.91	2.83	3.503 (8)	132
N00*F*—H00*H*···Br03^{v}	0.91	2.81	3.496 (9)	133
N00*F*—H00*G*···Br04^{iii}	0.91	2.55	3.438 (8)	166
N00*E*—H00*F*···Br05	0.91	2.44	3.340 (8)	168
N00*E*—H00*E*···Br0*A*	0.91	2.99	3.585 (8)	125
N00*E*—H00*E*···Br04^{ii}	0.91	2.93	3.438 (9)	117
N00*E*—H00*E*···Br03	0.91	2.66	3.333 (8)	131
N00*E*—H00*D*···Br08	0.91	2.64	3.463 (8)	151
N00*D*—H00*C*···Br07^{iv}	0.91	2.66	3.477 (7)	150
N00*D*—H00*B*···Br09	0.91	2.96	3.555 (8)	125
N00*D*—H00*B*···Br06^{v}	0.91	2.93	3.478 (8)	120
N00*D*—H00*B*···Br05^{iv}	0.91	2.67	3.326 (7)	130
N00*D*—H00*A*···Br03	0.91	2.45	3.344 (7)	166

Symmetry codes: (ii) −*x*+1, −*y*+2, −*z*+1; (iii) *x*, *y*−1, *z*; (iv) *x*+1, *y*, *z*; (v) −*x*+1, −*y*+1, −*z*+1.

Table S6. Hydrogen-bond geometry (Å, °) for **$(DPA)_5Pb_2Br_9$** at 273 K.

D—H···*A*	*D*—H	H···*A*	*D*···*A*	*D*—H···*A*
N5—H5*E*···Br9	0.89	2.63	3.349 (19)	139
N5—H5*D*···Br6	0.89	2.81	3.519 (18)	137
N5—H5*D*···Br3^{ii}	0.89	3.07	3.510 (17)	113
N5—H5*C*···Br4^{ii}	0.89	2.87	3.396 (15)	120
N5—H5*D*···Br3^{ii}	0.89	3.07	3.510 (17)	113
C25—H25*A*···Br9	0.97	2.99	3.570 (12)	120
N4—H4*F*···Br3^{iv}	0.89	2.79	3.495 (14)	137
N4—H4*E*···Br7^{v}	0.89	3.13	3.852 (13)	139
N4—H4*E*···Br6^{v}	0.89	2.81	3.469 (13)	132
N4—H4*D*···Br8^{v}	0.89	3.00	3.423 (12)	111
N4—H4*D*···Br2^{iv}	0.89	3.03	3.832 (14)	151
N3—H3*F*···Br7^{vi}	0.89	3.02	3.893 (15)	167
N3—H3*E*···Br8	0.89	2.99	3.737 (14)	143
N3—H3*E*···Br1^{ii}	0.89	2.99	3.441 (12)	114
N3—H3*D*···Br4^{ii}	0.89	2.64	3.430 (14)	149
C12—H12*B*···Br9	0.97	3.01	3.951 (17)	164
C12—H12*A*···Br8^{vi}	0.97	3.12	3.782 (15)	127
N2—H2*F*···Br2	0.89	2.81	3.605 (14)	150

N2—H2*E*···Br1	0.89	2.71	3.488 (13)	147
N2—H2*D*···Br7	0.89	2.85	3.607 (13)	144
N2—H2*D*···Br1^{i}	0.89	3.09	3.620 (14)	120
N1—H1*C*···Br7^{vii}	0.89 (1)	2.73 (2)	3.495 (12)	144 (2)
N1—H1*B*···Br2^{vii}	0.89 (1)	2.90 (2)	3.430 (15)	120 (2)
C5—H5*B*···Br2^{vii}	0.97	3.12	3.715 (14)	121

Symmetry codes: (i) −*x*+1, −*y*+1, −*z*+1; (ii) *x*−1, *y*, *z*; (iv) *x*−1, *y*−1, *z*; (v) *x*, *y*−1, *z*; (vi) −*x*, −*y*+1, −*z*+1; (vii) −*x*+1, −*y*+2, −*z*+1.

Table S7. Hydrogen-bond geometry (Å, °) for **(DPA)$_4$AgBiBr$_8$** at 150 K.

D—H···*A*	*D*—H	H···*A*	*D*···*A*	*D*—H···*A*
C7—H7*A*···Br1	0.99	2.87	3.77 (4)	151
N2—H2*C*···Br2^{i}	0.91	2.86	3.70 (2)	154
N2—H2*B*···Br3^{iii}	0.91	2.84	3.45 (2)	126
N2—H2*B*···Br1^{iii}	0.91	3.05	3.71 (2)	131
N2—H2*A*···Br4	0.91	2.66	3.43 (2)	144
N1—H1*E*···Br3^{iv}	0.91	2.72	3.56 (3)	154
N1—H1*D*···Br4^{ii}	0.91	2.52	3.34 (3)	150
N1—H1*D*···Br2^{i}	0.91	3.25	3.58 (3)	104
N1—H1*D*···Br1^{iv}	0.91	3.14	3.55 (3)	109
N1—H1*C*···Br2^{iv}	0.91	3.01	3.65 (3)	129
C1—H1*B*···Br1^{i}	0.99	2.97	3.88 (4)	154

Symmetry codes: (i) −*x*+1, −*y*+1, −*z*+1; (ii) −*x*+2, −*y*+2, −*z*+1; (iii) −*x*+1, −*y*+2, −*z*+1; (iv) *x*+1, *y*, *z*.

Table S8. Crystal data and structure refinements for **(DPA)$_5$Pb$_2$Br$_9$** and **(DPA)$_4$AgBiBr$_8$** at high temperature phase.

	**(DPA)$_5$Pb$_2$Br$_9$** (310 K)	**(DPA)$_4$AgBiBr$_8$** (375 K)
Empirical formula	$C_{25}H_{69}Br_9N_5Pb_2$	$C_{20}AgBiBr_8N_8$
Formula weight	1573.42	1308.41
Crystal system	Triclinic	Orthorhombic
Space group	$P\bar{1}$	*Cmmm*
a (Å)	11.996(2)	8.774(10)
b (Å)	13.422(2)	27.47(3)
c (Å)	17.477(3)	8.771(9)
α (°)	72.658(4)	90
β (°)	70.158(4)	90
γ (°)	77.179(4)	90
Volume(Å^3)	2503.6(8)	2114(4)
Z	2	2

Radiation type	MoK\a	MoK\a
Absorption correction	Multi-scan	Multi-scan
D_{calc} /g cm^{-3}	2.087	2.056
F (000)	1466	1172
GOF	1.054	1.244
$R_1[I > 2\sigma(I)]$	0.0605	0.1110
$wR2[I > 2\sigma(I)]$	0.1824	0.3235

Table S9. The reported space group and band gap of Ruddlesden−Popper AgBiBr- and AgBiI-based materials (n=1).

Formula	Space group	Band gap (eV)	Ref
$(BA)_4AgBiBr_8$	$C2/m$ (278 K)<br>$P2_1/c$ (100 K)	2.5	4
$(BA)_4AgBiBr_8$	*Bmab*	2.65	5
$(PA)_4AgBiBr_8$	$C2/m$	2.41	6
$(PA)_2CsAgBiBr_7$	$P2_1/m$	2.32	6
$(BDA)_4AgBiBr_8$	$P\bar{1}$	2.43	6
$(OcA)_4AgBiBr_8$	$P2/m$	2.45	6
$(DPA)_4AgBiBr_8$	$P\bar{1}$ (150 K)<br>*Cmmm* (375 K)	2.44	this work
$(IPA)_4AgBiI_8$	$P\bar{1}$	1.87	7
$(3\text{-}IPA)_4AgBiI_8$	$P\bar{1}$	1.87	7
$(4\text{-}AMP)_4AgBiI_8{\cdot}H_2O$	$C2_1/c$	2.1	8
$(4\text{-}APP)_4AgBiI_8{\cdot}H_2O$	$C2/c$	2.1	8

Appendix S1. Acronyms arranged in top-down order in Table S9.

BA: Butylammonium
PA: Propylammonium
BDA: Butane-1,4-diammonium
OcA: Octylammonium
DPA: 2,2-dimethylpropan-1-aminium
IPA: 3-iodopropan-1-aminium
3-IPA: 3-Iodopropylammonium
4-AMP: 4-(Ammoniomethyl) piperidinium
4-APP: 4-Ammoniumpiperidine

Reference:

(1) Kresse, G.; Furthmüller, J. Efficiency of ab-initio total energy calculations for metals and semiconductors using a plane-wave basis set. *Comput. Mater. Sci.* **1996**, *6*, 15-50.

(2) Perdew, J. P.; Ernzerhof, M.; Burke, K. Rationale for mixing exact exchange with density functional approximations. *J. Chem. Phys.* **1996**, *105*, 9982.

(3) Blöchl, P. E. Projector augmented-wave method. *Phys. Rev. B* **1994**, *50*,

17953-17979.

(4) Connor, B. A.; Leppert, L.; Smith, M. D.; Neaton, J. B.; Karunadasa, H. I. Layered Halide Double Perovskites: Dimensional Reduction of Cs2AgBiBr6. *J. Am. Chem. Soc.* **2018**, *140*, 5235-5240.

(5) McClure, E. T.; McCormick, A. P.; Woodward, P. M. Four Lead-free Layered Double Perovskites with the n = 1 Ruddlesden-Popper Structure. *Inorg. Chem.* **2020**, *59*, 6010-6017.

(6) Mao, L.; Teicher, S. M. L.; Stoumpos, C. C.; Kennard, R. M.; DeCrescent, R. A.; Wu, G.; Schuller, J. A.; Chabinyc, M. L.; Cheetham, A. K.; Seshadri, R. Chemical and Structural Diversity of Hybrid Layered Double Perovskite Halides. *J. Am. Chem. Soc.* **2019**, *141*, 19099-19109.

(7) Yao, Y.; Kou, B.; Peng, Y.; Wu, Z.; Li, L.; Wang, S.; Zhang, X.; Liu, X.; Luo, J. (C3H9NI)4AgBiI8: a direct-bandgap layered double perovskite based on a short-chain spacer cation for light absorption. *Chem. Commun. (Cambridge, U. K.)* **2020**, *56*, 3206-3209.

(8) Lassoued, M. S.; Bi, L.-Y.; Wu, Z.; Zhou, G.; Zheng, Y.-Z. Piperidine-induced Switching of the direct band gaps of Ag(i)/Bi(iii) bimetallic iodide double perovskites. *J. Mater. Chem. C* **2020**, *8*, 5349-5354.
